# Interactions between *Bacillus thuringiensis* and selected plant extracts for sustainable management of *Phthorimaea absoluta*

**DOI:** 10.1038/s41598-024-60140-4

**Published:** 2024-04-23

**Authors:** Terry A. Ochieng, Komivi S. Akutse, Inusa J. Ajene, Dora C. Kilalo, Maina Muiru, Fathiya M. Khamis

**Affiliations:** 1 International Centre of Insect Physiology and Ecology (icipe), P. O. Box 30772-00100, Nairobi, Kenya; 2 College of Agriculture and Veterinary Sciences, University of Nairobi, P. O. Box 30197-00199, Nairobi, Kenya; 3 Unit for Environmental Sciences and Management, North-West University, Potchefstroom, 2520 South Africa

**Keywords:** Microbial insecticide, South American tomato pinworm, Biorational control option, Neem, Garlic, Synergistic interaction, Gut microbiome, Biological techniques, Ecology, Microbiology, Molecular biology, Zoology, Ecology, Environmental sciences

## Abstract

*Phthorimaea absoluta* is a global constraint to tomato production and can cause up to 100% yield loss. Farmers heavily rely on synthetic pesticides to manage this pest. However, these pesticides are detrimental to human, animal, and environmental health. Therefore, exploring eco-friendly, sustainable Integrated Pest Management approaches, including biopesticides as potential alternatives, is of paramount importance. In this context, the present study (i) evaluated the efficacy of 10 *Bacillus thuringiensis* isolates, neem, garlic, and fenugreek; (ii) assessed the interactions between the most potent plant extracts and *B. thuringiensis* isolates, and (iii) evaluated the gut microbial diversity due to the treatments for the development of novel formulations against *P. absoluta*. Neem recorded the highest mortality of 93.79 ± 3.12% with an LT_50_ value of 1.21 ± 0.24 days, Bt HD263 induced 91.3 ± 3.68% mortality with LT_50_ of 2.63 ± 0.11 days, compared to both Bt 43 and fenugreek that caused < 50% mortality. Larval mortality was further enhanced to 99 ± 1.04% when Bt HD263 and neem were combined. Furthermore, the microbiome analyses showed that Klebsiella, *Escherichia* and *Enterobacter* had the highest abundance in all treatments with Klebsiella being the most abundant. In addition, a shift in the abundance of the bacterial genera due to the treatments was observed. Our findings showed that neem, garlic, and Bt HD263 could effectively control *P. absoluta* and be integrated into IPM programs after validation by field efficacy trials.

## Introduction

Tomato (*Solanum lycopersicum* Mill.) (Solanaceae) is the most widespread and consumed vegetable fruit globally^1^. Tomatoes have substantial medicinal, nutritional and economic values. For instance, the fruits contain carotenoid lycopene which is important in protection against chronic ailments^2^. The crop is well suited to different cropping systems with short maturity periods^3,4^. In Kenya, tomato is extensively cultivated and is an important source of income for small-holder farmers, foreign exchange earnings, contributes to GDP, and crucial provision of raw materials to the manufacturing industries^5^. Tomato crop constitutes approximately 14% and 7% of the entire Kenyan vegetable and horticultural sector, respectively^6^.

Despite the various benefits of tomato, its production and productivity have not kept pace with the immensely growing demand due to abiotic and biotic constraints. For instance, annual economic loss in Kenya and sub-Saharan Africa (SSA) is estimated at approximately US$ 59.3 and US$ 791.5 million, respectively^5,7^. Arthropod pests and diseases have been considered among the key biotic factors that have led to overreliance and indiscriminate use of chemical pesticides in Africa^8^. Recently, the invasive South American tomato pinworm, *Phthorimaea absoluta* (Meyrick) (Lepidoptera: Gelechiidae), has emerged as the biggest threat to tomato production in many African countries. The pest’s primary host plant is tomato, while other members of the family Solanaceae are alternative hosts of *P. absoluta*. The aggressive and cryptic nature of *P. absoluta* makes its management very difficult, while its short life cycle increases the rapid spread of the pest populations in most invaded African countries^9^. Furthermore, adults have high reproductive potential, with one female laying up to 300 creamy-coloured eggs and can produce up to 10–12 generations annually^10^. Larvae, which are characterized by their cryptic nature, are the most damaging life stages of all parts of tomato plants^11^. Larval instars feed by mining the mesophyll tissue of the leaf and creating irregular leaf mines, while mature larvae drop to the soil where they produce a thin, silky cocoon to transform into prepupae^12,13^. In case of severe infestation, larvae consume all the leaf mesophyll tissue leaving behind skeletonized leaves and a large amount of frass^14^. They also attack the fruits, making entry points for other pathogens causing fruit rot. These injuries and interruption of photosynthesis reduce the quality of fruits and yield, thus loss of lucrative export markets^7^.

Currently, synthetic pesticides are heavily used by growers as the main management method against *P. absoluta*. About 96.5% of tomato farmers in Kenya use chemicals, with some active substances being highly toxic to humans, the environment and biodiversity^7,15^. The misuse of broad-spectrum chemical pesticides also presents several shortcomings such as the development of resistance, non-target effects on pest’s natural enemies, and limited efficacy of the pesticides due to the cryptic nature of *P. absoluta*^16–18^. For example, it has been reported that *P. absoluta* has developed resistance to organophosphates, parathyroids, spinosyns, avermectins, indoxacarb and diamides^19–22^. Therefore, there is a strong need to develop more effective, safer, and sustainable alternatives to manage *P. absoluta* in an integrated approach in various solanaceous production systems.

Global demand for biopesticides is growing at an estimated rate of 15% annually compared to 3% for synthetic chemicals as a result of pressure from regulatory bodies and awareness of the adverse impacts of synthetic pesticides^23,24^. Although botanicals and entomopathogens/microbials are gaining momentum in pest management^25^, biopesticide use is still low and estimated at 3.0 billion USD, about 5% of the world’s pesticide market^26^. Entomopathogens are increasingly used as biopesticides against insect pests, and several entomopathogenic fungi and endophytes have been reported to be effective against *P. absoluta*^26^. Additionally, *Bacillus thuringiensis* has been shown to be an effective biorational alternative to applying chemical pesticides in *P. absoluta* management^27^. *Bacillus thuringiensis*, as a pathogen of insects, has high efficacy, specificity, and safety with no negative impacts on human and animal health and the ecosystem^28^.

Therefore, there is a need to increase the biopesticide portfolio by exploiting other safer biological products against major agricultural pests, including *P. absoluta*. *Bacillus thuringiensis* and botanicals such as neem, garlic, and fenugreek, which are known to be non-toxic, have been found effective against several pest species^23,29^ and could, therefore, be explored in the sustainable management of *P. absoluta*.

In this context, the present study screened 10 isolates of *B. thuringiensis*, evaluated the efficacy of neem, garlic and fenugreek extracts against 2nd instars of *P. absoluta* and evaluated the effects of the most effective *B. thuringiensis* isolates and botanicals on the pest gut microbiome for sustainable, integrated management of *P. absoluta*.

## Results

### Pathogenicity and virulence of*** Bacillus thuringiensis*** against 2nd instar of*** Phthorimaea absoluta***

**Figure 1:**
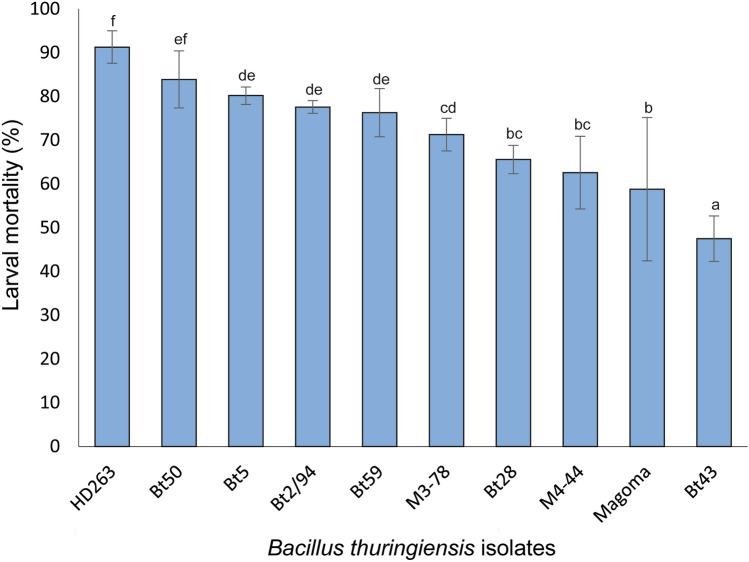
Pathogenicity of the various *Bacillus thuringiensis* isolates to 2^nd^ instar larvae of *Phthorimaea absoluta.*

The pathogenicity of the 10 *B. thuringiensis* strains screened against the 2nd instar of *P. absoluta* differed significantly (χ^2^ = 318.46, df = 9, *P* < 0.001) among the treatments (Fig. 1). Although all the Bt isolates were found pathogenic to the pest (> 50% larval mortality), only Bt 43 caused a mortality < 50% (Fig. 1). HD263 outperformed all the isolates by causing 91.3% mortality, followed by Bt 50 (83.9%), Bt 5 (80.2%) and Bt2/94 (77.6%) (Fig. 1).

The lethal time to 50% mortality caused by Bt 5 was the fastest (2.48 ± 0.19 days), followed by HD263 (2.63 ± 0.1 days) (Table 1). Although Bt 2/94 caused higher mortality than Bt 59, their LT_50_ values did not differ significantly, and similar trends were observed with M3-78 (6.6 ± 0.3 days) and M4-44 (6.23 ± 0.2 days) that recorded the lowest killing speed (knockdown effect) (Table 1).

**Table 1:** Lethal time (LT_50_) values caused by the various *Bacillus thuringiensis* isolates.

*B. thuringiensis* isolates	Mortality (%)	LT50 values (Days)
HD 263	91.3	2.6 ± 0.1
Bt 50	83.9	3.6 ± 0.1
Bt 5	80.2	2.5 ± 0.2
Bt29/4	77.6	4.2 ± 0.1
Magoma	58.8	3.0 ± 0.1
Bt 59	76.3	6.6 ± 0.3
Bt 28	65.6	5.7 ± 0.1
M3-78	71.3	6.6 ± 0.3
M4-44	62.6	6.2 ± 0.2

### Efficacy of neem, garlic and fenugreek extracts against 2nd instar of* Phthorimaea absoluta*

**Figure 2:**
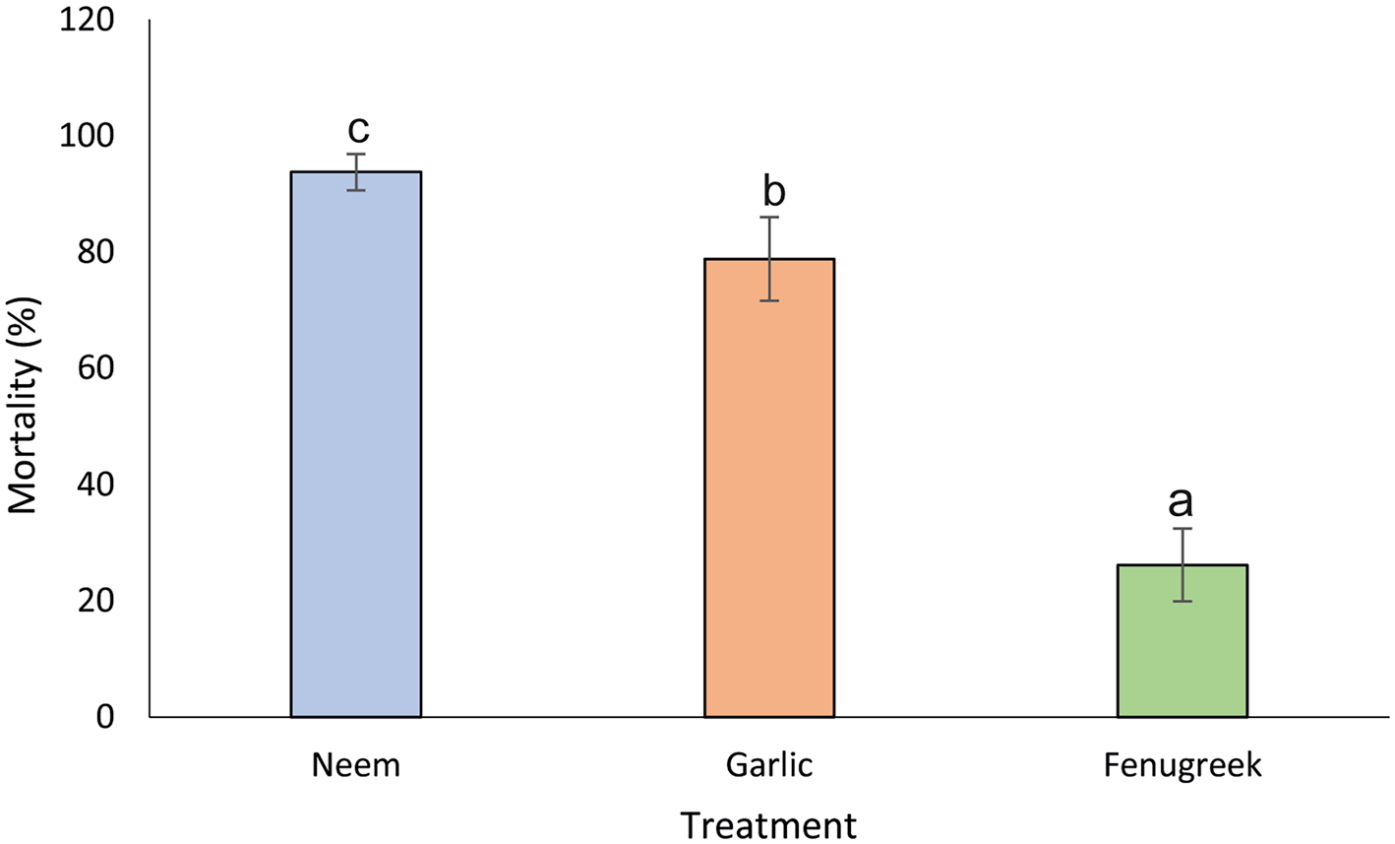
Effects of neem, garlic and fenugreek on 2nd instar of *Phthorimaea absoluta.*

Efficacy of the various tested extracts varied significantly (χ^2^ = 473.98, df = 2, *P* < 0.001) among the treatments (Fig. 2). All the three botanical extracts were pathogenic to *P. absoluta* larvae, with neem recording the highest mortality (93.8%) followed by garlic (78.8%), while fenugreek recorded the least mortality (26.25%) to larvae (Fig. 2). Additionally, neem extract application generated the lowest LT_50_ (1.21 ± 0.24 days) compared to 2.03 ± 0.2 days for garlic, while fenugreek did not kill half of the exposed larval populations.

### Effects of interaction between the potent* Bacillus thuringiensis* isolates and botanicals on the 2nd instar of *Phthorimaea absoluta*

**Figure 3:**
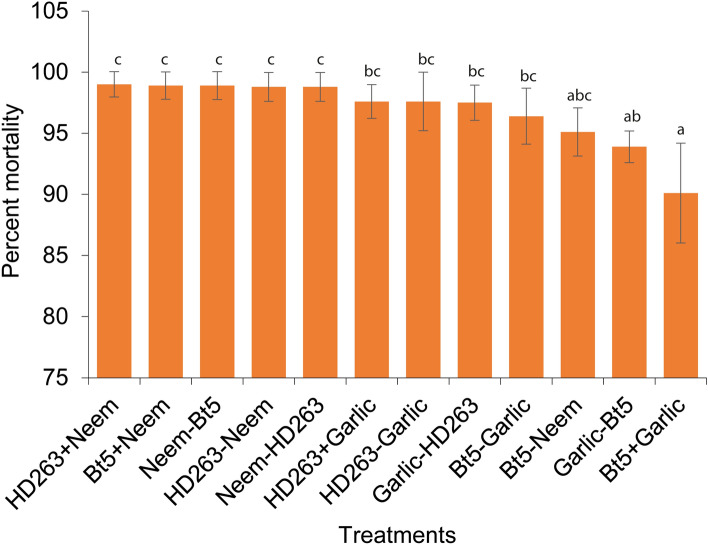
Effects of interaction between the potent *Bacillus thuringiensis* isolates (HD263 and Bt 5) and botanicals (Neem and Garlic) on the second instar of *Phthorimaea absoluta* in simultaneous (HD263 + Neem, Bt5 + Neem, HD263 + Garlic and Bt5 + Garlic) and sequential (Neem-Bt5, HD263-Neem, Neem-HD263, HD263-Garlic, Garlic-HD263, Bt5-Garlic and Garlic-Bt5) combinations.

Significant interaction was observed among the treatments (χ^2^ = 88.912, df = 11, *P* < 0.001) when combining *B. thuringiensis* isolates (HD263 and Bt 5) and plant (neem and garlic) extracts against 2^nd^ instar larvae of *P. absoluta* through sequential and simultaneous releases (Fig. 3). All the treatments/combinations recorded larval mortality of at least 90% (Fig. 3). Simultaneous exposure of HD263 + Neem (99%) recorded the highest mortality compared to the other 11 interaction treatments, while Bt5 + Garlic had the lowest mortality (90.1%) (Fig. 3). Bt5 + Neem and HD263 + Garlic lethality to larvae reached 98.9% and 97.6%, respectively, and were not significantly different from HD263 + Neem (99%) (Fig. 3). On the other hand, sequential releases of neem and Bt5, HD263 and neem, neem and HD263, HD263 and garlic, garlic and HD263, Bt5 and garlic, Bt5 and neem, and garlic and Bt5 induced mortality rates of 98.9%, 98.8%, 98.8%, 97.6%, 97.5%, 96.4%, 95.1%, and 93.9%, respectively (Fig. 3). All the simultaneous and sequentially combined treatments recorded > 90% mortality, with greatly enhanced knockdown effect or high killing speed that ranged between 0.04 and 1.84 days, as compared to when both the Bt and botanicals were screened solely (Fig. 3; Table 2).

**Table 2:** LT_50_ values of the interactions between the potent *Bacillus thuringiensis* and botanicals against second instar larvae of *Phthorimaea absoluta* in simultaneous (HD263 + Neem, Bt5 + Neem, HD263 + Garlic and Bt5 + Garlic) and sequential (Neem-Bt5, HD263-Neem, Neem-HD263, HD263-Garlic, Garlic-HD263, Bt5-Garlic and Garlic-Bt5) combinations.

Treatments	Mortality (%)	LT_50_ values (Days)
T_1-_ Neem Extract + HD 263	99	0.2 ± 1.1
T_2-_ Neem Extract → HD 263	98.8	0.1 ± 1.8
T_3-_ HD 263 → Neem extract	98.8	1.2 ± 0.1
T_4-_ Garlic Extract + HD 263	97.6	1.3 ± 0.1
T_5-_ Garlic Extract → HD 263	97.5	1.8 ± 0.1
T_6-_ HD 263 → Garlic Extract	97.6	0.5 ± 0.2
T_7-_ Neem Extract + Bt 5	98.9	0.5 ± 0.2
T_8-_ Neem Extract → Bt 5	98.9	0.6 ± 0.1
T_9-_ Bt 5 → Neem Extract	95.1	0.6 ± 0.2
T_10-_ Bt 5 + Garlic Extract	90.1	0.1 ± 0.7
T_11-_ Bt 5 → Garlic Extract	96.4	0.4 ± 0.2
T_12-_Garlic Extract → Bt 5	93.9	0.9 ± 0.2

### Gut microbial bacterial diversity of 2nd instar ***Phthorimaea absoluta ***larvae exposed to potent*** B. thuringiensis*** isolates and botanicals

**Figure 4:**
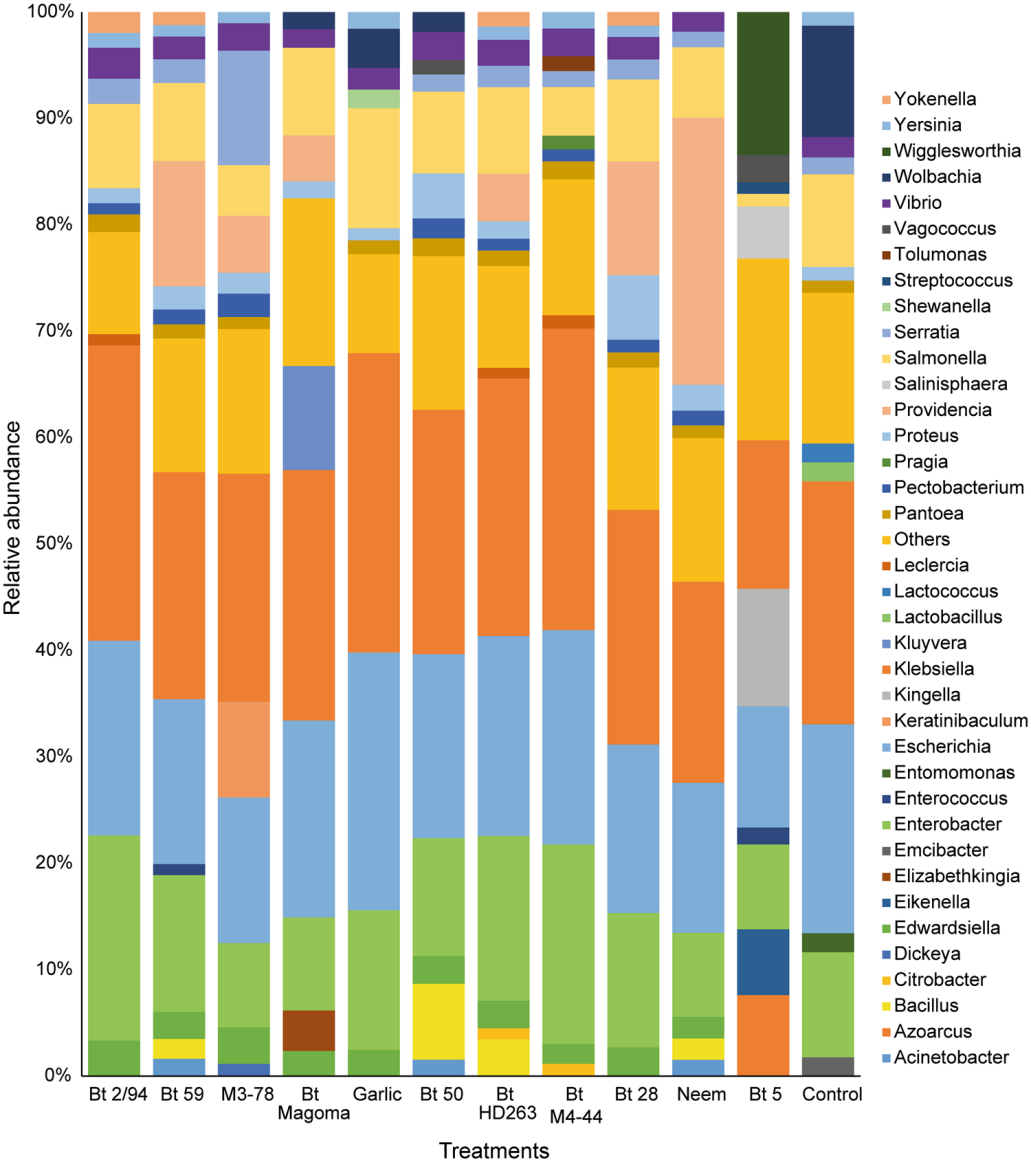
Relative abundance of the gut microbiome of 2nd instar *Phthorimaea absoluta* larvae exposed to *B. thuringiensis* (Bt) isolates, neem, and garlic.

The comparative analyses of the composition and cumulative abundance of the *P. absoluta* 2^nd^ instar larvae treated with *B. thuringiensis* isolates, neem and garlic showed that *Klebsiella* was the most abundant bacterial genera in all treatments with larvae treated with Bt 43 having the highest abundance of *Klebsiella* (28.26%) and larvae treated with Bt 5 having the least abundance (13.97%). A high abundance of *Escherichia* and *Enterobacter* was also observed across all the treatments, ranging from 7.98 to 23.66% (Fig. 4).

**Figure 5:**
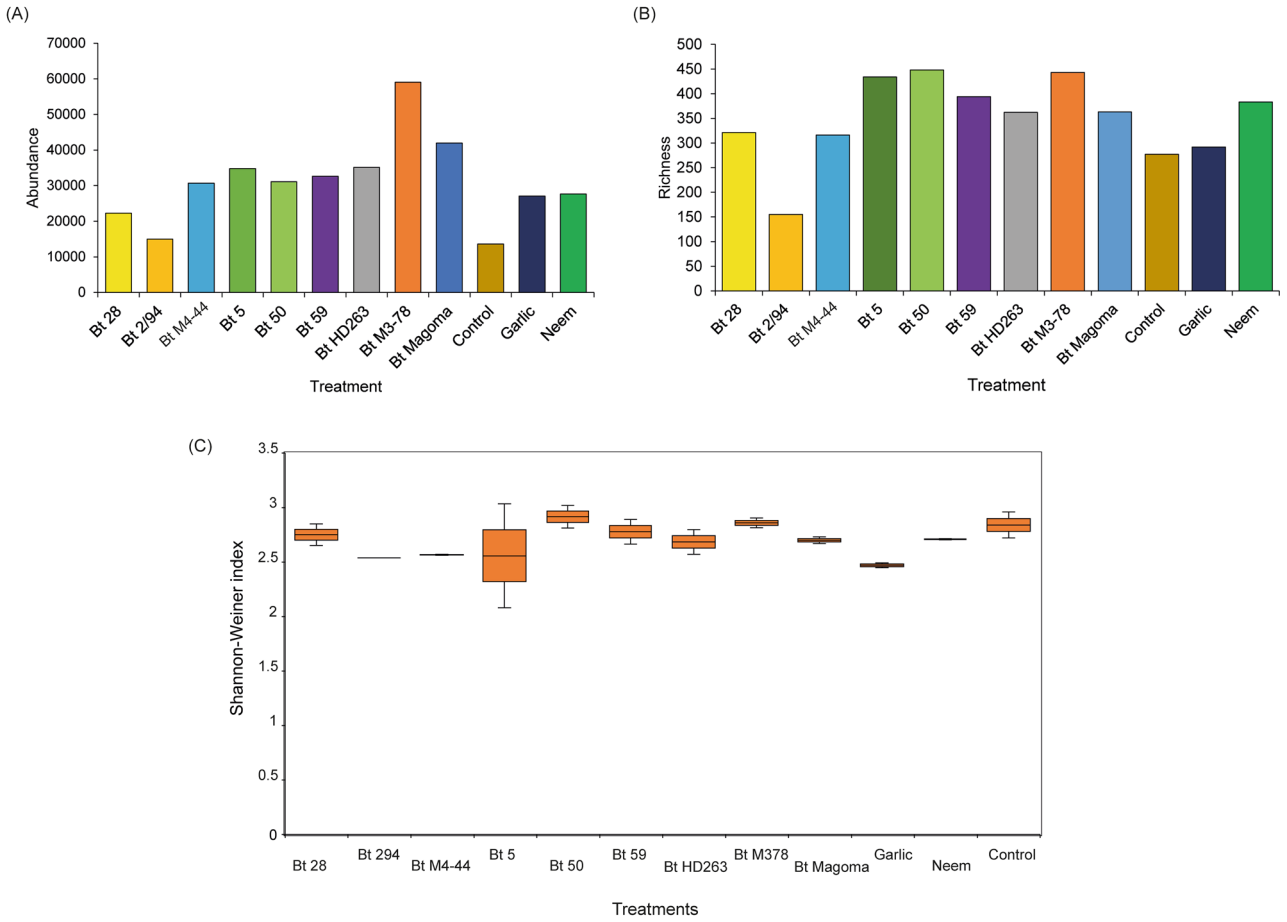
Alpha diversity of the gut microbiome of 2nd instar *Phthorimaea absoluta* larvae exposed to *B. thuringiensis* (Bt) isolates, neem, and garlic showing (**A**) Evenness, (**B**) Abundance and (**C**) Shannon diversity.

The analysis of the gut microbial bacterial diversity among the 12 treatments revealed that *P. absoluta* larvae treated with Bt M3-78 had the highest microbial abundance, and the least was observed in the control treatment (Fig. 5A). The highest species richness was observed in *P. absoluta* larvae treated with Bt 50, followed by Bt M3-78 and Bt 5, while the lowest was observed in larvae treated with Bt 2/94 (Fig. 5B). *Phthorimaea absoluta* larvae treated with Bt 5 had the highest Shannon diversity between all treatments, while *P. absoluta* larvae treated with garlic had the least Shannon diversity (Fig. 5C).

**Figure 6:**
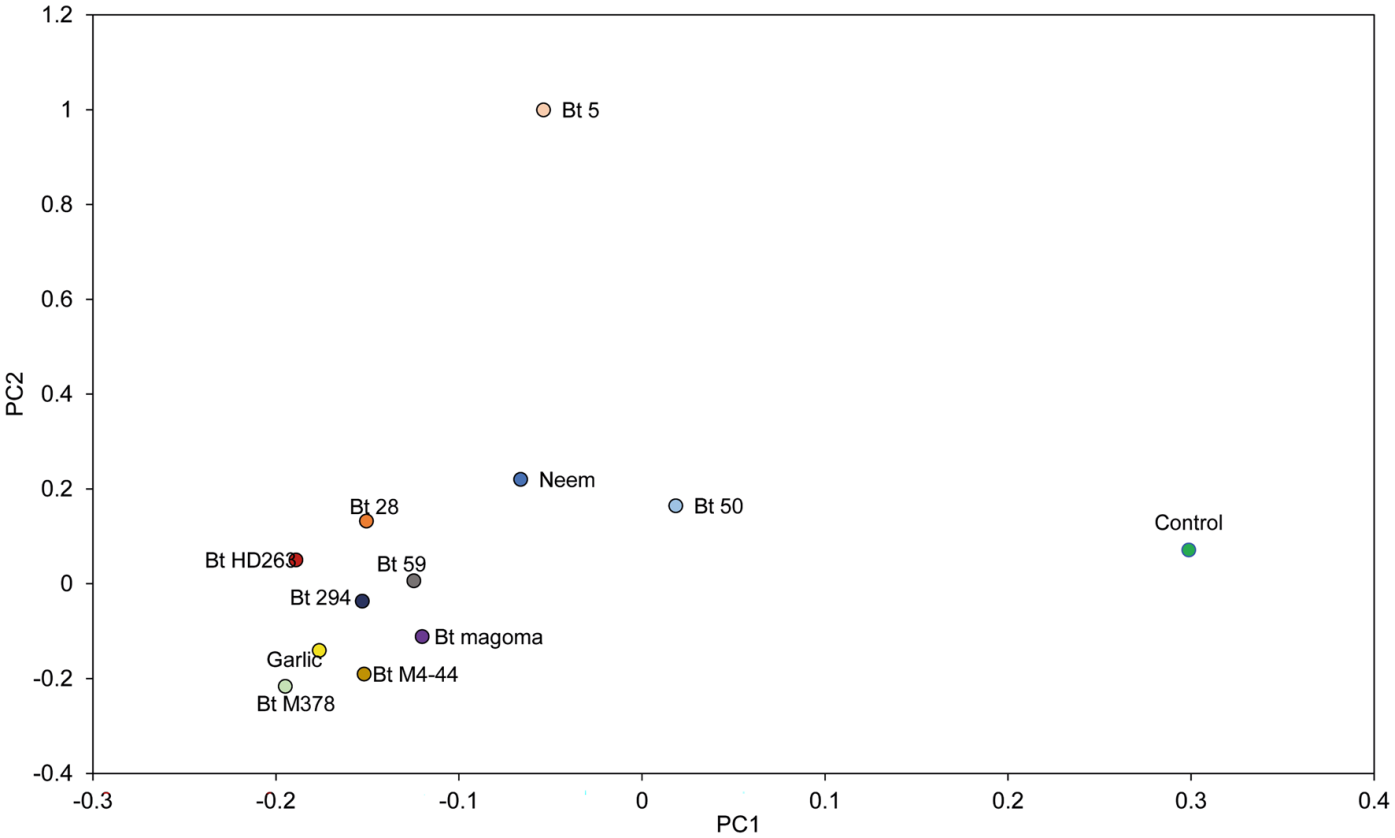
Interpopulation diversity of the gut microbiome of 2^nd^ instar *Phthorimaea absoluta* larvae exposed to *B. thuringiensis* (Bt) isolates, neem, and garlic estimated using the Bray–Curtis dissimilarity index.

The beta diversity revealed a relatively high diversity between the treated larvae and the untreated control with three clusters (Fig. 6). There was low diversity between the different treatments, with the closest interpopulation distance observed between larvae treated with Bt 59 and larvae treated with Bt 294. The highest interpopulation distance between the treatments was observed between larvae treated with Bt 5, which clustered separately from the other treatments and larvae treated with Bt M378, indicating a shift in the microbial communities after treatment (Fig. 6).

## Discussion

This study evaluated the pathogenicity and virulence of some selected *B. thuringiensis* isolates from *icipe’s* repository of entomopathogens, and botanicals (neem, garlic, and fenugreek extracts) against *P. absoluta* 2nd instar larvae in laboratory bioassays through separate and combined treatments assessments, for enhancing sustainable management programs of this pest.

Our study demonstrated high pathogenicity and virulence of *B. thuringiensis* isolates against *P. bsoluta* 2nd instar larvae*,* evidenced by significantly high larval mortality rates and low lethal times. However, pathogenicity varied among the various *B. thuringiensis* strains used. Among the 10 isolates screened, nine isolates recorded higher than 50% mortality, with the HD263 isolate outperforming the other isolates screened. These findings concur with previous reports, which showed high mortality (78–91%) performance of *P. absoluta* treated with *B. thuringiensis*^30^. Similarly, high susceptibility of the first and third instars of *P. absoluta* to *B. thuringiensis* has previously been reported, whereby leaflet damage due to *P. absoluta* instars was reduced by up to 70% upon treatment with *B. thuringiensis*^31,32^. Furthermore, our study showed that *B. thuringiensis* alone can effectively control *P. absoluta,* as shown by the HD263 isolate, which killed 91.3% of the tested larval population. This has also been demonstrated in studies by González-Cabrera et al.^31^, who concluded that *B. thuringiensis* can effectively control *P. absoluta* even with no additives. Our study also showed that the *B. thuringiensis* isolates HD263, Bt 50, Bt 5 and magoma killed 50% of the exposed larvae within 3 days of feeding on *B. thuringiensis* treated leaves, confirming the LT_50_ values reported by Alsaedi et al.^33^, who observed the greatest mortality on the third day after larvae were fed on leaves treated with *B. thuringiensis* suspension.

In addition to *B. thuringiensis* isolates, our evaluation of the efficacy of three botanical (neem, garlic and fenugreek) extracts against 2nd instar of *P. absoluta* revealed the exposed larval populations were susceptible to the selected botanicals, causing > 50% mortality in two out of the three botanicals screened. Among the botanicals, neem and garlic extracts caused the highest mortality (93.8% and 78.8%, respectively), while fenugreek (26.25%) was the least lethal botanical. Hence, neem and garlic extracts are very potent botanical candidates for sustainable management of *P. absoluta*, however field trials should be performed to validate these laboratory findings. Suppressed feeding and repellence of larvae were observed in our study, as exposed *P. absoluta* larval populations avoided the neem and garlic-treated tomato leaves after a short period of exposure and feeding. Our findings agree with previous studies, which showed neem and garlic were effective natural alternatives to synthetic chemicals in controlling pests such as *Earias vittella* Fabricius, 1794 (Lepidoptera: Nolidae)^34^, *Toxoptera citricida* Kirkaldy, 1907 (Hemiptera: Aphididae)^35^, *Spodoptera littoralis* Biosduval, 1833 (Lepidoptera: Noctuidae)^2^*, Spodoptera eridania* Stoll, 1781 (Lepidoptera: Noctuidae)^36^ and *Sesamia cretica* Lederer, 1857 (Lepidoptera: Noctuidae)^37^, by causing repellence, repugnance and anti-feedant effects. These results could, therefore, aid in developing new botanical pesticide formulations to sustainably control *P. absoluta* in solanaceous cropping systems*.*

Integrated management strategies such as the application of *B. thuringiensis* in combination with botanicals such as neem to prevent the potential development of resistance to *B. thuringiensis* in insect pests presents a sustainable, eco-friendly approach to pest management^38^. Our study evaluated combined treatment assessments of the potent botanicals and *B. thuringiensis* isolates against *P. absoluta* for sustainable management of the pest. The treatments’ potency was enhanced when the treatments were combined and subjected to *P. absoluta* 2nd instar larvae. Larval mortality was significantly higher in both simultaneous and sequential combinations of the two biocontrol agents. All the treatments, both sequential and simultaneous caused > 90% larval mortality. The knockdown effect was also greatly enhanced, LT_50_ values ranging between 0.04 and 1.84 days, compared to sole exposure of both Bt isolates and botanicals to the pest. Simultaneous assessments of HD263 and neem, and Bt 5 and neem outperformed other treatments. Moreover, sequential treatments of Bt 5 and neem, neem and Bt 5, HD263 and neem, and neem and HD263 recorded mortality of about 99%, indicating synergistic or additive effects. Previous studies on *Plodia interpunctella* Guenée, 1845 (Lepidoptera: Pyralidae) and *Helicoverpa armigera* Hübner, 1808 (Lepidoptera: Noctuidae) larvae exposed to combined treatments of Bt toxins and azadirachtin also revealed synergistic and additive interactions, an indication of complementary action when compared to sole exposures^38,39^. Therefore, the synergetic effects of both biocontrol agents against the pest make the combined treatments suitable for integration in an Integrated Pest Management (IPM) program for sustainable control of the pest.

The analyses of the effect of the potent *B. thuringiensis* isolates and botanicals on the gut microbiome of second-instar *P. absoluta* larvae revealed the high abundance of *Klebsiella*, *Escherichia* and *Enterobacter* in both the untreated and treated *P. absoluta* 2^nd^ instar larvae. Albeit minimal, there was a reduction in the abundance of these bacteria and a shift in the microbial diversity upon treatment with the *B. thuringiensis* isolates and the botanicals suggesting gut microbial repression by the treatments. The reduction in key gut bacteria has also been observed in *Trichoplusia ni* Hübner, 1800 (Lepidoptera: Noctuidae), where treatment with neem volatiles reduced Enterobacteria transcript amplification^40^. Furthermore, our study found that *P. absoluta* 2nd instar larvae treated with the botanicals (neem and garlic) had lower microbial abundance than most of the *B. thuringiensis* isolates*.* This finding agrees with previous studies, which showed that higher susceptibility of neem was associated with lower enterobacterial load in *T. ni*^40^ and higher susceptibility to *B. thuringiensis* was associated with higher enterobacterial loads in *Spodoptera exigua* Hübner, 1808 (Lepidoptera: Noctuidae)*,* probably due to the mode of action of *B. thuringiensis*, which involves the cry proteins^41,42^.

With these significant findings, it is also important to indicate that this study was performed under controlled laboratory conditions. Therefore, it is hypothesized that the results under field conditions might differ, calling for further field trials for validation of our findings. Furthermore, future studies are warranted to assess the dose responses of the various individual and combination treatments against the various larval instars and to determine the best formulations of both the potent *B. thuringiensis* and botanicals for effective management of *P. absoluta.* It is also essential to validate these laboratory results under semi-field and field conditions for better recommendations, as well as to assess the impact of our treatments on biodiversity, including pollinators and natural enemies associated to *P. absoluta*, to guide future adoption of these combined formulations in the integrated management of the pest under different cropping systems. Moreover, further studies are warranted to explore the key resistance genes expression involved in the treatment of the larvae with *B. thuringiensis* isolates and the botanicals in association with the observed gut bacterial shifts.

## Conclusion

Our findings demonstrate the efficacy of *B. thuringiensis*, neem, and garlic extracts, solely or in combination as potential natural products for sustainable management of *P. absoluta* in IPM systems. Individual exposure of *B. thuringiensis* isolates to *P. absoluta* revealed high susceptibility of the pest and could potentially be used to develop effective, safe and affordable microbial pesticides for the management of *P. absoluta*. Additionally, the results from our research showed that individual exposure of botanicals, neem and garlic extracts, has significant potential in controlling *P. absoluta*. Developing neem and garlic into biopesticides could therefore offer a better alternative to synthetic pesticides, enabling safer control of *P. absoluta* pest populations. In addition, farmers could also be trained on making their own innovative neem and garlic-based products to reduce the cost of pest control. Moreover, the results from our interaction study further demonstrate the potential of biorational pesticides which combined *B. thuringiensis* with botanicals with additive or synergetic effects against *P. absoluta*. The performance of the potent *B. thuringiensis* isolates was greatly enhanced when combined with neem and garlic extract, whether simultaneously or sequentially. We could hypothesize that combining these treatments could be more economical compared to *B. thuringiensis* toxic protein isolation. Also, it reduces the chances of the pest’s resistance to either of these control agents. Thus, our identified potent *B. thuringiensis* and botanical candidates could be developed into natural products that can effectively and sustainably control *P. absoluta* under IPM programs as safer alternatives to chemical pesticides without any detrimental health risks.

## Materials and methods

### Rearing of *Phthorimaea absoluta*

*Phthorimaea absoluta* colony used in this study was obtained from Animal Rearing and Quarantine Unit of the International Centre of Insect Physiology and Ecology (*icipe*). The colony rearing followed the procedure described by Agbessenou et al.^24^. The initial colony was established from 120 wild larvae and adults (1:2 male to female ratio) collected from infested tomato fruits and leaves from Isiolo County (0°21′20.2896″N 37°34′59.898″E, 300 m.a.s.l.) in Kenya in 2019. The colony was rejuvenated after every three months through infusion with freshly collected infested tomato leaves from the field to avoid in-breeding. The insects were reared on Moneymaker variety tomato plants in well-ventilated sleeved Perspex cages (Perspex International, Darwen Lancashire, UK) measuring (50 cm × 50 cm × 45 cm) up to 5–8 generations to ensure colony stability prior to bioassays. At least 20 pairs of newly emerged *P. absoluta* adults were exposed to four healthy potted tomato plants (approximately 20-day-old seedlings) per Perspex cage for oviposition and maintained at 25–27 °C temperature, 50–70% relative humidity and 12L: 12D photoperiod. A solution containing 10% honey was placed ad libitum at the top of each one of the cages as source of nutrition for the moths^24^. After 24 h of exposure to the adult females, the plants were transferred to netted wooden cages measuring 50 cm × 50 cm × 60 cm until the eggs hatched into larvae. Three days after larval emergence, leaves containing larvae were pruned and transferred into sterile plastic containers measuring 8 cm × 15 cm × 21 cm. Paper towel lining was placed for absorption of excess moisture with netting infused lids for enabling ventilation. Second instar larvae, creamy in color were selected for the experiments. The remaining larvae were fed on fresh tomato leaves until they pupated to maintain the colony. The pupae were then transferred to separate clean plastic containers using fine camel hairbrush for adult emergence. *Phthorimaea absoluta* colony was monitored daily and maintained at the temperature of 25 ± 2 °C, 50–70% RH and 12L:12D photoperiod.

### Preparation of biopesticide inoculum

#### Bacillus thuringiensis

Ten *B. thuringiensis* isolates were obtained from *icipe*’s Arthropod Pathology Unit germplasm repository and used in this study (Table 3). Culture and suspension preparation of *B. thuringiensis* were based on the procedure described by^43^. The isolates were maintained on Luria Bertani (LB) media containing final ingredients concentrations of Tryptone 10 g, yeast extract 5 g, NaCl 10 g, Agar technical 15 g, and 1000 ml of sterile distilled water. The LB broth was autoclaved for 90 min at 120 °C, dispensed into 40 Petri dishes. The isolates were then cultured by plating on the media using the streaking method, and the Petri dishes were then incubated for 24 h at a temperature of 37 °C. A single colony of each *B. thuringiensis* strain was transferred to 50 ml Luria Bertani liquid media and incubated for 72 h at 37 °C in an incubator shaker (New Brunswick™ Innova® 44, Eppendorf, Germany). Crystal formation was observed using phase contrast microscopy by smearing slides with each isolate and adding a basic solution (Safranin), which was then rinsed with running water. The slides were covered with coverslips and immersed in glycerol oil to ensure clear viewing under the microscope. The crystal expression was observed under a microscope at a magnification of X100. Observation was done under a Light Microscope (Leica Microsystems, UK, Ltd), at X100 magnification for crystal/toxin expression. The cells were harvested by centrifugation at 3214 rpm for 10 min. The pellets obtained were rinsed once with cold 1 M NaCl and further rinsed twice with cold sterile distilled water. To the final wet pellet (spore‐crystal mixture), 20 ml of sterile distilled water was added, where the colony forming units were determined, adjusted at the working concentration of 1 × 10^8^ CFU/ml, and stored at − 20 °C until commencement of the bioassays.

**Table 3:** List and identity of *Bacillus thuringiensis* isolates used in the bioassays against *Phthorimaea absoluta.*

Country	*B. thuringiensis* isolates	Host	*icipe* accession number	Year
USA	HD 263	Soil	Kurstaki (commercial isolate)	1994
Kenya	Bt 50	Grain	ICIPE 131	1993
	Bt 5	Grain	ICIPE 230	2004
	Bt 2/94	Soil	ICIPE 142	1993
	Bt Magoma	Grain	ICIPE 203	2004
	M4-44	Soil	ICIPE 193	1994
	M3-78	Soil	ICIPE 227	2002
	Bt 59	Grain	ICIPE 205	2004
	Bt 28	Grain	ICIPE 204	2004
	Bt 43	Grain	ICIPE 202	2004

### Garlic aqueous extract

Fresh locally grown organic garlic bulbs of uniform size were selected and obtained from a local market in Nairobi, Kenya (Naivas Supermarket—Kasarani, Nairobi, Kenya), and stored at room temperature prior to the bioassays. Randomly selected samples of garlic bulbs were rinsed in sterile distilled water and ground in a ratio of 1 g:1 ml to make an aqueous extract of 100% concentration, using a sterile pestle and mortar. The homogenate aqueous solution was then centrifuged at 10,000 rpm, and the supernatant was collected after filtration through a sterile 0.22 µm pore filter paper. Fresh extracts were prepared just before conducting the experiment to ensure maximum efficiency.

### Neem nimbecidine and fenugreek

Neem suspension (Nimbecidine) and fenugreek powder were purchased from an Agrovet in Nairobi, Kenya (Table S1). Nimbecidine is a natural botanical pesticide composed of azadirachtin (0.003%) and neem oil (90.57%) against insect pests, mites, and soil nematodes in a wide range of crops.

### Pathogenicity of *Bacillus thuringiensis* strains against 2nd instar of* Phthorimaea absoluta*

Evaluation of the 10 isolates of *B. thuringiensis* against second instar of *P. absoluta* was performed using spore-crystal mixtures at the concentration of 1 × 10^8^ CFU/ml. Pathogenicity was evaluated following the method described by O’Callaghan et al.^44^. Forty clean fresh tomato leaves were surface sterilized using 70% ethanol for 1 min 30 s and rinsed twice with sterile distilled water. The leaves were air-dried and sprayed with 3 ml of each isolate suspension using a hand sprayer. They were then dried in a laminar flow hood after treatment. Twenty 2nd instar larvae were carefully transferred onto whole treated leaf in a Petri dish using soft camel brush, and each Petri dish is considered as a replication. The procedure was used for all the 10 isolates. Each treatment had four replications in a completely randomized design and maintained at laboratory conditions of 25–27 °C temperature, 50–70% RH and 12L: 12D photoperiod, monitored daily for 8 days. Control experiment was also arranged following the same approach but treated with only sterile distilled water without any bacteria. The plates were sealed using parafilm, small holes made at the top for aeration and the larvae allowed to feed for 24 h, after which leaves were removed daily and replaced with fresh untreated leaves. Mortality data was recorded from each treatment daily for 7 days.

### Efficacy bioassays of neem, garlic, and fenugreek extracts against 2nd instar of *Phthorimaea absoluta*

The assessment was conducted following the methodology described by^45^. The bioassays were conducted to assess both contact toxicity and stomach poisoning (antifeedant) effects of the treatments on the 2nd instar of *P. absoluta*. Clean fresh tomato leaves were surface sterilized using 70% ethanol and rinsed twice with sterile distilled water. The leaves were sprayed with 3 ml concentrated solutions of neem (5 ml/20 L), garlic (100%) and fenugreek (100%) using a hand sprayer^44^; and were then air-dried under the laminar flow hood. Twenty 2^nd^ instar larvae of *P. absoluta* were also sprayed and pat-dried. then carefully transferred using soft camel brush onto a treated tomato leaflet, in a Petri-dish. Each treatment was replicated four times in a completely randomized design. Control treatment was done using sterile distilled water under the same conditions. The plates were sealed using parafilm, small holes made at the top for aeration and the larvae allowed to feed. The Petri dishes were maintained at 25 °C ± 2 °C temperature, 50–70% RH and 12L:12D photoperiod during data collection in the laboratory. Mortality data of the larvae was recorded daily for 7 days; where the leaves were removed daily and replaced with fresh untreated leaves.

### Interaction between the most potent botanicals and* Bacillus thuringiensis* isolates

From the above experiments, two *B. thuringiensis* isolates (HD263 and Bt 5), neem and garlic extracts were found very effective against *P. absoluta* larvae. The interactions between these potent biocontrol candidates using sole/single, sequential and simultaneous combinations were evaluated following the methodology described by Akutse et al.^8^. Thirteen treatments were defined through simultaneous and sequential releases of the larvae (Table S2): T1 (larvae exposed to neem extract + HD263 simultaneously for 24 h); T2 (larvae sequentially exposed to neem extract for the first 24 h followed by exposure to HD263 for the next 24 h); T3 (larvae sequentially exposed to HD263 for the first 24 h, and to neem extract for the next 24 h); T4 (larvae simultaneously exposed to HD263 + garlic extract for 24 h); T5 (larvae sequentially exposed to garlic extract for 24 h, followed by exposure to HD263 for another 24 h); T6 (larvae sequentially exposed to HD263 for 24 h, followed by exposure to garlic extract for another 24 h); T7 (larvae simultaneously exposed to neem extract + Bt 5 for 24 h); T8 (larvae sequentially exposed to neem extract for the first 24 h, followed by exposure to Bt 5 for the next 24 h); T9 (larvae sequentially exposed to Bt 5 for the first 24 h, and to neem extract for the next 24 h); T10 (larvae simultaneously exposed to Bt 5 + garlic extract for 24 h); T11 (larvae sequentially exposed to Bt 5 for the first 24 h, and to garlic extract for the next 24 h); T12 (larvae sequentially exposed to garlic extract for the first 24 h, and to Bt 5 for the next 24 h) and T13 (control treatments). Using a hand sprayer, 48 tomato leaves were sprayed with 3 ml of the various treatments (4 leaflets per treatment) accordingly and air-dried in the laminar flow hood prior to their exposure to the larvae. The larvae were also sprayed and pat-dried. During the simultaneous and sequential application of the botanical treatments. One treated leaf was placed in a Petri dish, and soft camel brush used to transfer 20 larvae into each Petri dish of the various treatments. Each treatment was replicated four times in a completely randomized design and maintained at laboratory conditions of 25 ± 2 °C temperature, 50–70% RH and 12L:12D photoperiod. The plates were sealed using parafilm, small holes made at the top for aeration and the larvae allowed to feed as described above. Leaves were removed daily and replaced with fresh untreated ones. Larval mortality was daily assessed and recorded from each treatment for 7 days.

### Effects of *Bacillus thuringiensis* isolates, neem, and garlic on *Phthorimaea absoluta* 2nd instar gut microbiota

#### Sample collection

Evaluation of the nine potent isolates of *B. thuringiensis* against second instar of *P. absoluta* was performed using spore-crystal mixtures of moderately higher concentration (1 × 10^8^). Dead larvae fed on *Bt* and plant extracts treated leaves were collected and dipped in 3% sodium hypochlorite for 3 s, rinsed using sterile distilled water thrice to free them from microbial contaminants. The larvae were then stored at − 80 °C for gut microbiota analyses.

#### Genomic DNA extraction and 16S rRNA sequencing

DNA was extracted from the samples obtained from the experiment using the Isolate II Genomic DNA kit (Bioline, London, UK). DNA quality and concentration were determined using a Nanodrop 2000/2000c Spectrophotometer (Thermo Fischer Scientific, Wilmington, USA). DNA extracts within A_260nm_/A_280nm_ ratio of 1.8–2.0 were eluted to 50 µl final volume. Sequencing libraries were prepared using the Oxford Nanopore Technology (ONT) 16S barcoding kit (SQK-16S024). The full-length (~ 1480 bp) bacterial 16S rRNA was sequenced on an ONT MK1C sequencing device using FLO-MIN106 flow cells. Sequencing was run for four hours with live base calling performed using Albacore tool (v2.3.4) on the MinKNOW (v20.10.3) software on the ONT cloud. FastQ files that resulted generated from sequencing were fed to EPI2ME (v3.5.4-8056458) software. The "What's in my Pot" (FASTQ-WIMP (v3.2.1) workflow in EPI2ME was used in the identification and classification of bacterial species in real-time and assigned taxonomy at the genus level using the NCBI reference database. The sequence data from this study was submitted to the NCBI database under the BioProject ID: PRJNA1052641 (https://dataview.ncbi.nlm.nih.gov/object/PRJNA1052641?reviewer=vkjph4j67vteua6233qj05jt0e).

### Data analysis

Larval mortality data were corrected using Abbott’s formula to eliminate natural mortality. Corrected mortality was tested for normality and arcsine transformed before subjection to analyses. Mortality data was analyzed using one-way analysis of variance (ANOVA) in a General Linear Model (GLM) for a binomial distribution using the logit link function. Significantly different means (*P* < 0.05) were separated using Student–Newman–Keuls (SNK) test. Lethal times taken to score 50% mortality of the exposed insect population were estimated for the treatments that recorded over 50% mortality of the 2nd instar larvae of *P. absoluta*. Time-mortality data were analyzed with GLM using the function “dose. *p*” from the MASS library in R statistical software, to generate LT_50_ estimates, alongside slopes of regression curves. Each replication was analyzed using GLM analysis. The resultant LT_50_ values and their respective slopes were further run through ANOVA to generate means. The means were separated using Students Newman Keuls (SNK) test at 95% confidence interval (*P* < 0.05). All data analyses were conducted using R (version 4.1.2) statistical software package^46^.

### Bacterial diversity statistics

A stacked bar plot was used to compare direct quantitative abundances at the genus level for each treatment. The most abundant taxa from each sample were selected based on a minimum abundance cut-off 1%. Due to the highly diversified community, bacterial species with cumulative reads counts below 1% were collapsed to form “others” for visualization of better taxonomic patterns. Alpha diversity metrics (richness, evenness, and Shannon-Weiner index) were used to determine the bacterial diversity in each sample, and the Bray–Curtis dissimilarity index was computed to determine diversity in bacterial genera among the samples. Alpha and beta diversity statistics were computed using R (version 4.1.2) statistical software package^46^.

### Ethical approval

The experimental research and field studies on plants, including the collection of plant material, complied with relevant institutional, national, and international guidelines and legislation. The appropriate permissions and/or licenses for collection of plant or seed specimens were obtained for the study. All insect rearing, handling and experiments were performed using standard operating procedures at the *icipe* Animal Rearing and Quarantine Unit as approved by the National Commission of Science, Technology and Innovations, Kenya (License No: NACOSTI/P/20/4253). This article does not contain any studies with human participants performed by any of the authors.

### Supplementary Information

Supplementary Information.

## Data Availability

All other relevant data are within the paper and supplementary materials. Sequences generated from this study were deposited in the GenBank database (www.ncbi.nlm.nih.gov/genbank) under the BioProject: PRJNA1052641 (https://dataview.ncbi.nlm.nih.gov/object/PRJNA1052641?reviewer=vkjph4j67vteua6233qj05jt0e).
