# Supplementary material for: Interactions between Bacillus thuringiensis and selected plant extracts for sustainable management of Phthorimaea absoluta
Source: Sci Rep. 2024 Apr 23;14:9299. doi: 10.1038/s41598-024-60140-4 (PMC11039665; doi:10.1038/s41598-024-60140-4)
Supplement: Supplementary file 1 — Supplementary Information. [file 41598_2024_60140_MOESM1_ESM.docx]

**Interactions between *Bacillus thuringiensis* and selected plant extracts for sustainable management of *Phthorimaea absoluta***

Terry A. Ochieng^1,2^, Komivi S. Akutse^1,3*^, Inusa Jacob Ajene^1^, Dora Kilalo^2^, Maina Muiru^2^, Fathiya M. Khamis^1^

^1^International Centre of Insect Physiology and Ecology (*icipe*), P. O. Box 30772-00100, Nairobi, Kenya.

^2^University of Nairobi, College of Agriculture and Veterinary Sciences, P. O. Box 30197-00199, Nairobi, Kenya.

^3^Unit for Environmental Sciences and Management, North-West University, Potchefstroom, 2520, South Africa

*Corresponding author: [kakutse@icipe.org](mailto:kakutse@icipe.org)

**Table S1**. List of botanicals used in this study.

| **Botanical Pesticide** | **Concentration** | **Source of Pesticide** |
| --- | --- | --- |
| Neem Nimbecidine | 5mL/20L | Osho Company Limited |
| Garlic Extract | 1g/mL | Muthurwa Market (Nairobi, Kenya) |
| Fenugreek Powder | 1g/mL | Muthurwa Market (Nairobi, Kenya) |

**Table S2.** List of interaction treatments

| **Interaction Treatments** | |
| --- | --- |
| T_1-_ Neem Extract + HD 263 | T_7-_ Neem Extract + Bt 5 |
| T_2-_ Neem Extract HD 263 | T_8-_ Neem Extract Bt 5 |
| T_3-_ HD 263 Neem extract | T_9-_ Bt 5 Neem Extract |
| T_4-_ Garlic Extract + HD 263 | T_10-_ Bt 5 + Garlic Extract |
| T_5-_ Garlic Extract HD 263 | T_11-_ Bt 5 Garlic Extract |
| T_6-_ HD 263 Garlic Extract | T_12-_Garlic Extract Bt 5 |
